# Kinomic profiling of glioblastoma cells reveals PLCG1 as a target in restricted glucose

**DOI:** 10.1186/s40364-018-0136-9

**Published:** 2018-06-14

**Authors:** Kiera Walker, Nathaniel H. Boyd, Joshua C. Anderson, Christopher D. Willey, Anita B. Hjelmeland

**Affiliations:** 1 Department of Cell, Developmental and Integrative Biology, University of Alabama at Birmingham, Birmingham, AL 35294 USA; 2 Department of Radiation Oncology, University of Alabama at Birmingham, Birmingham, AL USA

**Keywords:** Glioblastoma, Tumor initiating cell, Cancer stem cell, PLCG1, Tumor microenvironment, Restricted glucose, Kinomics

## Abstract

**Background:**

For glioblastoma (GBM) treatments to be effective in vivo, understanding the effects of the tumor microenvironment is imperative. In traditional cell culture conditions, glucose concentrations do not model physiologic levels, nor the diminished concentrations found in tumor niches. We therefore sought to profile the differences in kinase activity in GBM cells cultured in restricted glucose to identify pathways that could be targeted with small molecule inhibitors.

**Methods:**

Using the PamStation12 platform, we examined the ability of GBM lysates from cells cultured in standard or low glucose conditions to phosphorylate 144 tyrosine and 144 serine/threonine peptides that correspond to known protein phosphorylation sites. Potential kinase targets were identified and validated using small molecule kinase inhibitors in GBM spheroid cultures.

**Results:**

Using results from two GBM patient-derived xenografts, we determined common changes to peptides derived from Phospholipase C, Gamma 1 (PLCG1) and Raf-1. Using PLC and Raf inhibitors, we found a significantly stronger growth inhibitory effect of the PLC inhibitor U73122 under restricted glucose conditions. In contrast, Raf inhibitors were significantly growth inhibitory regardless of the nutrient level tested.

**Conclusions:**

Together, our data demonstrate that kinase activity is altered in low glucose conditions and that kinomic profiling can assist with the identification of effective strategies to target GBM growth. Our data further suggest the importance of accurately modeling the tumor microenvironment to reproduce cancer cell signaling and develop drug screens for anti-cancer agents.

## Background

Glioblastoma (GBM) is a highly aggressive brain tumor and the most commonly occurring primary malignant glioma in adults, accounting for approximately 50% of all primary malignant brain tumor diagnoses [1–3]. Standard of care consists of surgical resection, concurrent radiation and chemotherapy, followed by adjuvant chemotherapy. While this treatment has extended the average survival to 14.6 months and increased 2 year survival to 17%, the overall prognosis still remains poor [4, 5]. GBM has proven difficult to treat due to tumor heterogeneity and the presence of tumor microenvironments such as low pH, oxygen, and nutrients [6–14]. Initially described as the Warburg Effect, tumor cells can activate alternative metabolic pathways for production of ATP and biomolecules to circumvent microenvironmental obstacles and fuel tumor growth [15]. Nutrient restriction is a modulator of the cellular metabolic state and can alter the kinase signaling pathways in the cell, with glucose playing a key role as a precursor for protein, nucleic acid, and lipid synthesis [9, 16–20].

Tyrosine kinase inhibition is a common modality in cancer treatment, as a myriad of components of the protein tyrosine kinase family have been recognized as proto-oncogenes [21]. Previous drugs developed to impede tyrosine kinase activity for cancer treatment have had limited success, as one of the major challenges is the presence or development of resistance to treatment with long-term use, such as acquired resistance to epidermal growth factor receptor (EGFR) inhibitors [21]. High-throughput profiling of kinase activity (kinomics) allows direct measurement of targetable activity, without the limitations of using genomic or other molecular surrogates. Paired with an unbiased prediction tool, kinomics has been utilized to help determine responders from non-responders, with the goal to improve drug efficacy by applying this technique to patient stratification [21]. The critical aspect of this technology is its ability to precisely measure the pertinent mechanism of action of a kinase inhibitor [21].

One important group of enzymes that may be altered by kinase activity during cancer progression are phospholipase C (PLC) family members, which serve as modulators of intracellular lipids and are involved in many cancer signaling cascades. Phospholipase C, gamma 1 (PLCG1) is most notably characterized in cancer by activation of cellular proliferation in response to growth factors such as epidermal growth factor receptor (EGFR) and platelet derived growth factor receptor (PDGFR), both common pathways altered in GBM. Elucidating how these kinase pathways change in response to local microenvironments during GBM progression will allow more directed approaches in treatment.

In this study, we sought to determine how kinase activity may be modulated by the tumor microenvironment in GBM, with the goal of identifying important pathways that could be targeted for cancer treatment. Utilizing kinase arrays, we were able to determine differences in peptide phosphorylation that are nutrient dependent and predict pathways important for GBM growth. Our experiments demonstrate the importance of accurately modeling the tumor microenvironment for drug screening.

## Methods

### Cell culture

Cells from dissociated GBM patient-derived xenografts (PDX) GBM14 and GBM456 were cultured at 37 °C in Dulbecco’s Modified Eagle’s Medium (DMEM)/F-12 50/50 with no phenol red, containing Gem21 NeuroPlex supplement w/o vitamin A, 1% Penicillin/Streptomycin, 1% Sodium Pyruvate, and 20 ng/mL each of recombinant human EGF and FGF basic 145aa. For kinomic assays, cells were plated from each cell line in high glucose media [Neurobasal-A Medium, 25 mM D-Glucose, 1% Penicillin/Streptomycin, 1% Sodium Pyruvate, 1% L-glutamine, 20 ng/mL hEGF and hFGF, B27] or low glucose media [high glucose media diluted 1:10 with Neurobasal-A supplemented medium without D-Glucose – final concentration of glucose is 450 mg/L or 2.5 mM] and incubated at 37° for a minimum of 3 days prior to harvest. Normal Human Astrocytes (NHA) were purchased from Lonza and cultured and treated in DMEM high glucose supplemented with 10% Fetal Bovine Serum, 1% N2 NeuroPlex, 3μg hEGF, and 1% Penicillin/Streptomycin. NHA cells were split and treated alongside the GBM PDX cells with the indicated inhibitors.

### Multiplex in vitro kinase assay

Kinomic profiling was performed in the UAB Kinome Core using standard methods [22–25]. Briefly, the protein lysates were extracted from the cells as described previously and quantified using a standard BCA assay with duplicates of each sample loaded at 15μg/well for tyrosine (PTK) assay or 2μg/well for serine/threonine (STK) assay onto the PamChips (PamGene International, ‘s-Hertogenbosch, The Netherlands). The in vitro kinase assay images were captured using the PamGene Evolve Software on the PamStation®12 platform and then images were imported to BioNavigator for raw primary analysis. Phosphorylation as measured by FITC intensity was captured multiple times during the course of the assay as the lysate is pumped across the array in cycles for the kinetic portion of the assay. During the kinetic portion, 50 ms exposures were used for image capture at the indicated cycle number. At the end of the reaction, the lysate and kinase assay reagents were rinsed off and multiple camera exposures (10, 20, 50, 100 ms) were integrated into a post wash slope, multiplied by 100 and Log2 transformed to compare and visualize signal in both low and high intensity peptides [21].

### Unsupervised clustering analysis

Unsupervised hierarchical clustering of both sample(column) and peptide(row) using Euclidian distance means-based hierarchal clustering method with complete linkage was performed within BioNavigator on all peptides to generate a clustered heatmap, with a heatmap colored by Signal change (from peptide mean across all replicates) and dendrogram trees to show similarity clustering. Additionally, to measure the strength of these relationships, a pvclust algorithm using R (v3.1.1) and Rstudio was used to generate approximately unbiased (au) and bootstrap probability (bp) values for each branch for high signal (> 7.0) peptides.

### Inhibitor treatment

Inhibitors were obtained from Selleck Chemicals and resuspended in DMSO to 10 mM stocks that were used via serial dilution to generate 1000× stocks for use at 1 μL/mL to generate the indicated final concentrations. After incubating in high and low glucose media for at least 3 days, GBM14 and GBM456 cells were split without using a dissociation reagent or Accutase was used to split NHA, into 96 well plates in triplicate at a density of 1000 cells/well in their corresponding high or low glucose media. Cells were allowed to recover for 24 h prior to drug or vehicle treatment. Cells were treated with the PLC inhibitor U73122 to a final concentration of 0.5, 2, and 4 μM or the Raf inhibitor AZ628 at a final concentration of 0.5 or 2 μM. Using Cell Titer Glo 2.0 reagent (Promega), growth of cells was measured using a luminometer after treatment with inhibitor at the indicated timepoints. This experiment was replicated a minimum of three times.

### Statistical analysis

For inhibitor treatments, a student’s t-test was used to compare the effects of treatment under restricted and standard glucose at the indicated time points with the indicated concentration of drug.

## Results

### Evaluation of the GBM Kinome in physiologic glucose

**Fig. 1:**
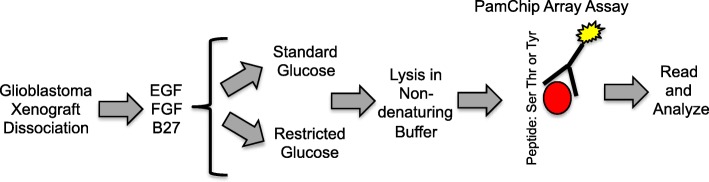
Overview of the kinomics experimental approach. The PamChip Array Assay permits analysis of peptides with Serine, Threonine, or Tyrosine phosphorylatable sites. We utilized the assay to measure kinase activity in lysates isolated from GBM cells cultured in low or standard glucose as outlined

**Fig. 2:**
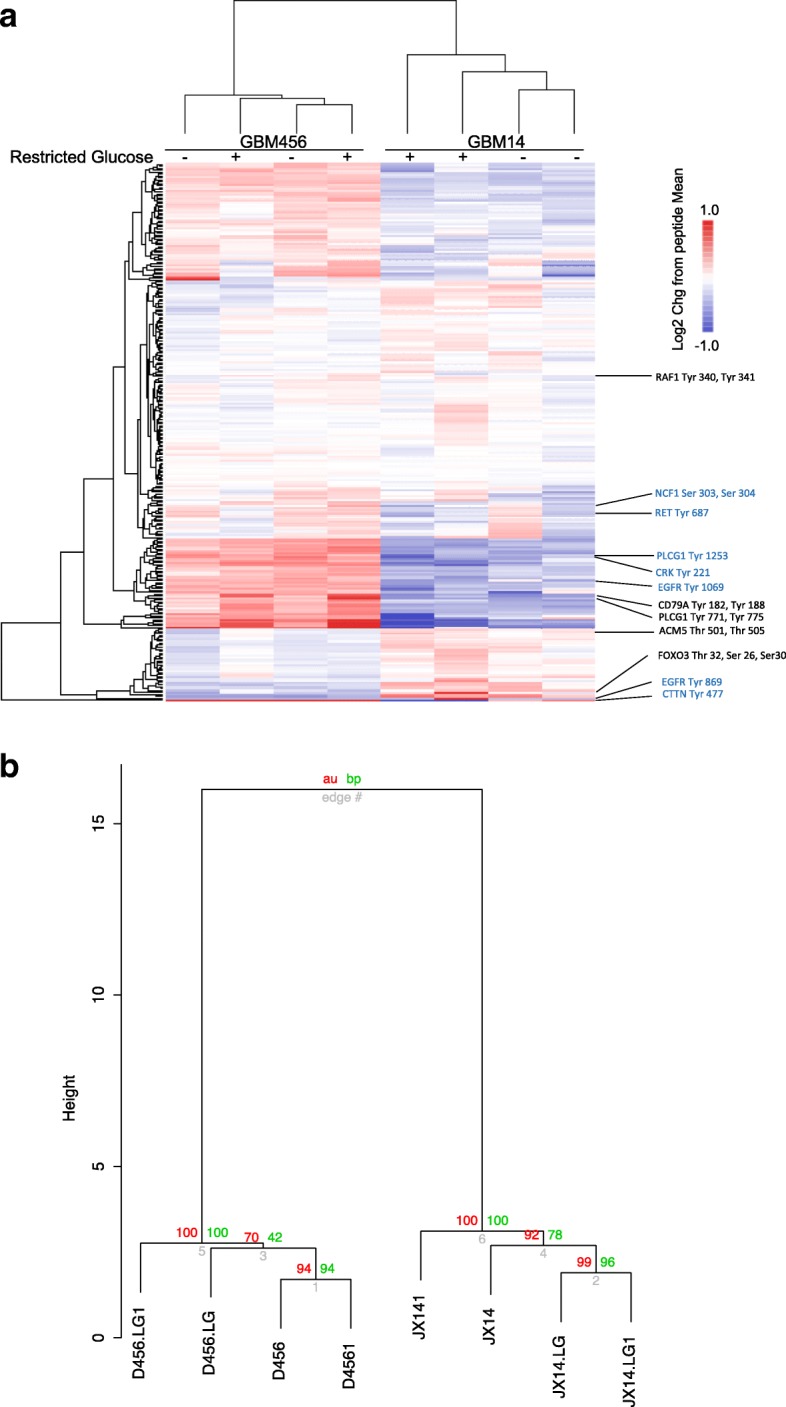
Heatmap of kinomic profiling with xenograft derived cells cultured in standard or restricted glucose. **a** Unsupervised clustering of kinomic data from cells isolated from two different GBM PDX of distinct subtype indicates grouping by xenograft followed by the treatment condition (standard or restricted glucose) (**b**). Scoring of dendrogram branching using Pvclust with unsupervised clustering (Euclidean/ward) of kinomic data having >7.0 average Log2 values with approximately unbiased (au) and bootstrap probabilities (bp) values superimposed. Nboot iterations = 1000

To better understand the GBM kinome in physiologically relevant tumor microenvironments, we cultured GBM cells in standard (25 mM) or low (2.5 mM) glucose (Fig. 1). GBM cells were obtained from dissociated PDX and subsequently cultured in the absence of serum but in the presence of EGF, FGF, and B27. These conditions have been shown to best recapitulate parental tumors [26]. We then performed kinomics on both serine/threonine and tyrosine kinase arrays to monitor phosphorylation of substrate peptides with known phosphorylatable target sequences. Following quality control examination of the phosphorylation curves, we performed an unsupervised hierarchical clustering analysis to determine how the kinomic signatures of high signal peptides clustered across all samples/conditions. Notably, the most obvious clustering was by xenograft origin (Fig. 2a). These data, and the scoring of the clustering strengths, suggest that any differences in the kinome due to changes in glucose levels do not override the intrinsic differences in the kinome of xenografts derived from different patients (Fig. 2a-b).

**Fig. 3:**
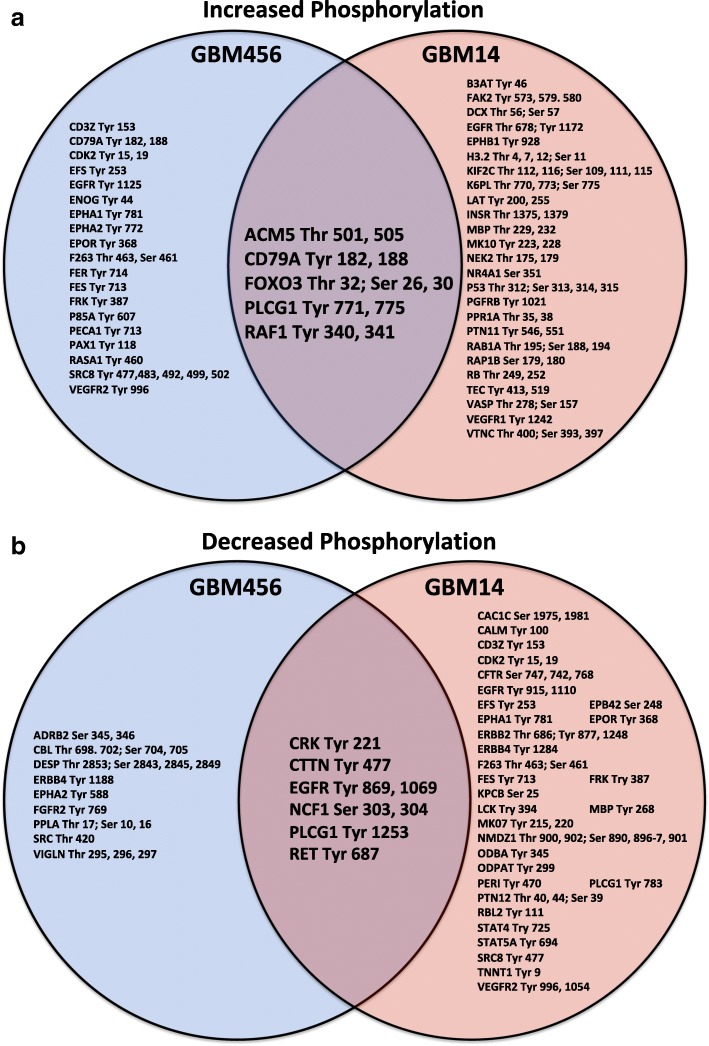
Proteins and phosphorylation sites corresponding to peptides differentially phosphorylated in restricted glucose. All peptides with a Log2 change > 0.3 (**a**) or < − 0.3 (**b**) when GBM456 (blue) or GBM14 (red) cells were cultured in low glucose in comparison to standard conditions are indicated. The Venn Diagram shows overlapping peptides in the center that have Log2 changes indicated above in both cell types. Peptides for which there was at least a > 0.5 Log2 change in one cell type and > 0.2 Log2 change in the other are also shown in the overlapping region in **a**. Peptides for which there was at least a < − 0.5 in one cell type and < − 0.2 Log2 change in the other are also shown in the overlapping region in **b**. The predominant common changes occur at tyrosine phosphorylation sites

We next sought to determine if there were common peptides that were differentially phosphorylated with low glucose culture between the two xenografts. We identified peptides that were uniquely regulated in either GBM456 or GBM14 cells with a Log2 change in restricted glucose greater than 0.3 (Fig. 3a) or less than − 0.3 (Fig. 3b). The phosphorylatable sites in the proteins that correspond to those in the individual peptides are indicated. Of note, the protein modification targets that were identified with culture in low glucose are predominately tyrosine phosphorylation sites.

A subset of peptides also displayed similar changes in phosphorylation in both GBM456 and GBM14. To identify peptides with common increases in phosphorylation, which are shown in both the labels to the right of the clustered heatmap (Fig. 2a) and in the overlapping regions of the Venn diagrams (Fig. 3a), the change was required to be in the same direction with a Log2 of at least 0.3 in both cell types or 0.5 in one cell type and 0.2 in the other. Using these criteria, five peptides were shown to have common increases in phosphorylation with restricted glucose exposure. These were peptides derived from regions of muscarinic acetylcholine receptor M5 (ACM5_498_510), cluster of differentiation CD79A (CD79A_181_193), Forkhead box protein O3 (FOXO3_25_37), phospholipase C, gamma 1 (PLCG1_764_776), and Raf-1 (RAF1_332_344). Among these, only the PLCG1 peptide demonstrated Log2 increases greater than 0.5 for both GBM456 and GBM14. These data suggested common increases in PLCG1 phosphorylation at tyrosine 771 and 775 in the presence of restricted glucose.

We also used a similar approach to identify peptides with common decreases in phosphorylation upon exposure to restricted glucose. Using the criteria of a Log2 change of at most − 0.3 in both cell types or − 0.5 in one cell type and − 0.2 in the other, six peptides were shown to have common decreases in phosphorylation (Fig. 3b). These peptides corresponded to regions of CRK (CRK_214_226), cortactin (SRC8_CHICK_470_482), epidermal growth factor receptor (EGFR_1062_107), neutrophil cytosol factor 1 (NCF1_296_308), PLCG1 (PLCG1_1246_1258), and RET (RET_680_692). Of these peptides, only the EGFR derived peptide demonstrated Log2 change of of at most -0.5 for both GBM456 and GBM14, but changes in PLCG1 and CRK had a Log2 of -0.4 or less in both cell types.

### Targeting restricted glucose mediated changes in phosphorylation

**Fig. 4:**
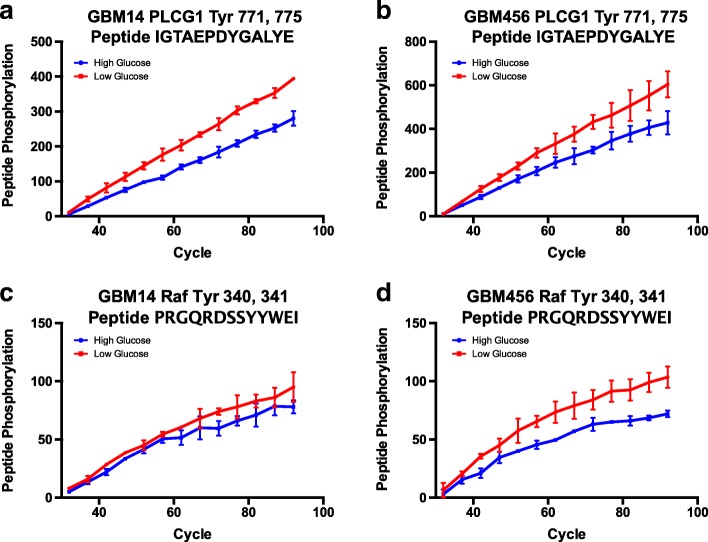
Kinetic curves for peptides corresponding to regions of PLCG1 and RAF1. Kinomic profiling demonstrated changes in the peptides that contain tyrosine phosphorylation sites corresponding to regions of PLCG1 (**a**, **b**) and Raf-1 (**c**, **d**). Representative curves for results from lysates isolated from GBM14 (**a**, **c**) or GBM456 (**b**, **d**) cells cultured in standard or restricted glucose with measurements over pumping cycles (time) are shown. Phosphorylation as measured by FITC intensity was captured at the indicated cycle number as the lysate was pumped across the array in cycles

**Fig. 5:**
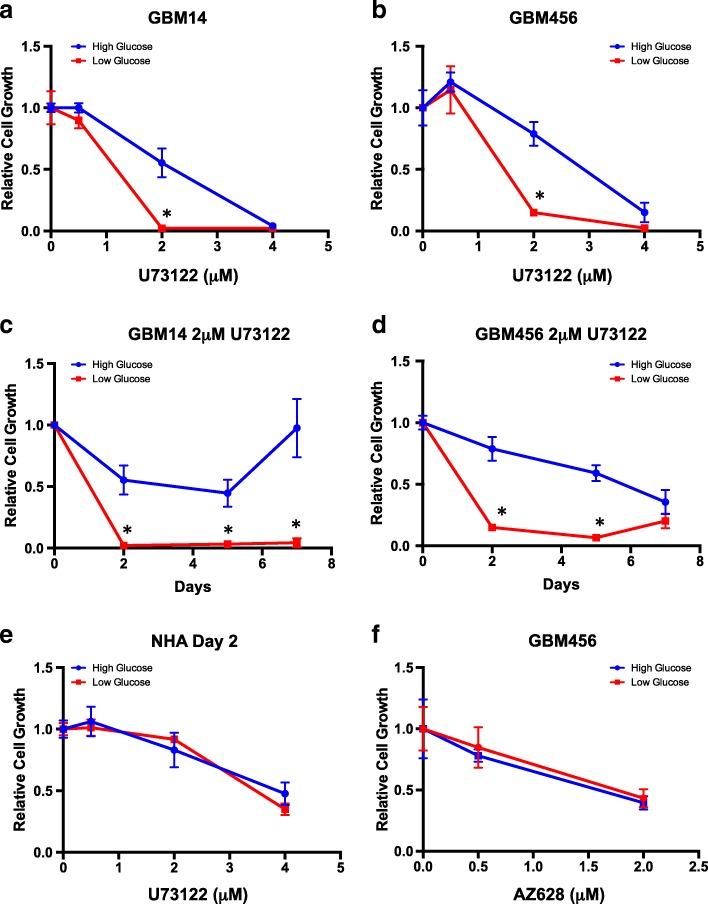
The efficacy of a PLC, but not Raf, inhibitor was increased when GBM cells were grown in restricted glucose. GBM cells isolated the indicated xenografts (**a-d**) or non-transformed human astrocytes (**e**) were cultured in standard or low glucose with or without U73122 or DMSO as a vehicle control. Data were normalized to the DMSO control of the appropriate glucose condition at the same time point. *, *p* < 0.05 with t-test comparison. **f** No significant difference in efficacy of the Raf inhibitor AZ628 was observed with changes in glucose levels in the media

As the data suggested increased phosphorylation at a subset of common targets among GBMs, we next considered whether inhibition of these targets could have distinct effects in low glucose. PLC and RAF small molecule inhibitors were commercially available, and we confirmed that the kinetic curves (captured over pumping cycles) for peptides PLCG1_764_776 and RAF1_332_344 demonstrated increased phosphorylation upon incubation with lysates from GBM cells exposed to low glucose in comparison to standard glucose (Fig. 4). The kinetic curves had the most consistent differential slope for the PLCG1 peptide, so we first tested the impact of increasing concentrations of the PLC inhibitor U73122. Using an ATP-based luminescent assay to screen for general changes in cell growth, the highest concentration of U73122 caused similar inhibition of proliferation under both standard/high and restricted/low glucose conditions. However, 2 uM of U73122 caused less than 50% growth inhibition in GBM PDX cells in standard cell culture conditions, whereas treatment in low glucose blocked GBM cell growth (Fig. 5a, b). A time course of growth effects similarly demonstrated that there was a significant increase in the efficacy of 2 uM of U73122 when cells were exposed to low glucose (Fig. 5c, d). This effect was not observed in non-neoplastic astrocytes (Fig. 5e). We also did not observe a significant difference in the efficacy of the RAF inhibitor AZ628 against GBM cells cultured in standard or restricted glucose (Fig. 5f). Together, these data indicate that small molecule inhibitor efficacy may differ depending on the level of glucose in the cell with a specific finding that PLC but not RAF inhibition was significantly different in restricted glucose.

## Discussion

We have investigated the impact of restricted glucose on the GBM kinome to develop a better understanding of how modeling of GBM microenvironments could improve our knowledge of tumor cell signaling and drug screening. Therapies identified via in vitro screens often fail in clinical trials. While this has been previously attributed to the now well recognized limitations of long-term passaged cell lines, we expect that a major contributor is also the failure to culture cells in vitro under conditions that best mimic the in vivo microenvironment. Improving in vitro model systems is therefore likely to be one cost-effective method to improve the translational potential of drug studies.

We found that the kinome of GBM cells is altered by culture in physiologic glucose in comparison to the standard cell culture conditions. Specific peptides for regions in PLCG1 and RAF1 with Tyr phosphorylation sites were more actively phosphorylated under low glucose conditions. Signaling pathways related to PLCG1 and RAF1 have been previously identified to be activated and/or mutated upon restricted glucose culture: AKT phosphorylation [27] and RAS mutation [28] increase in restricted glucose. While these data provide some validation of our approach, other peptide phosphorylation changes indicate alterations to pathways that have not been thoroughly investigated in GBMs. A peptide corresponding to ACM5 (CHRM5) was differentially phosphorylated in restricted glucose, but there are limited reports on the potential role of this muscarinic receptor in glioma. Data do suggest that acetylcholine can act through muscarinic receptors to induce proliferation of astrocytes and glioma cells [29, 30], potentially via the M3 receptors [31]. In contrast, activation of M2 receptors has been associated with decreased glioma growth [32, 33] and increased cell death [34], including in the tumor initiating cell subset [35]. Changes in phosphorylation to the peptide corresponding to regions of CD79A, the immunoglobulin-associated alpha chain of the B-cell antigen receptor complex, further suggest the importance of studying the impact of tumor microenvironments on immune system function. Thus, our findings suggest additional avenues of investigation to further explore the mechanisms through which restricted glucose impacts cancer growth.

PLCG1 is part of the gamma subclass of PLCs which exclusively contain SH2 and 3 domains, and the enzyme has been reported to be an important effector of activated RTKs such as EGFR and PDGF [36, 37]. The targeting and recruitment of this enzyme to activated RTKs requires the SH2 domain [38]. Therefore, the observed effects in the current study of increased phosphorylation of Tyr771 and Tyr775 in the context of low glucose could reasonably be attributed to increased RTK signaling, as these phosphorylation sites lie within the SH2 homology domain. Indeed, EGFR is predicted to be activated in low glucose when analyzing our results with Kinexus Kinase Predictor (data not shown). While EGFR inhibitors have thus far not led to significant improvements in GBM patient survival, these findings and literature do suggest that other low glucose activated or elevated upstream kinases could be targeted based on the kinomic array results.

Short term exposure to restricted nutrient conditions alters kinase activity in GBM but intrinsic differences in kinomes between patient samples remain: clustering of kinomic data was most strongly dependent on the xenograft rather than the glucose level. These data suggest that the different genetic alterations between tumors are likely to be the dominant force driving a kinomic signature. However, we did not compare the kinome of the GBM cells in distinct cell culture conditions to that in a portion of the non-dissociated tumor directly. The possibility remains that, even when glucose is at physiological levels, cell culture itself promotes kinomic shifts. By comparing parental tumor profiles to those in distinct cell culture conditions, we may identify the optimal in vitro conditions to mimic in vivo cell signals. Such optimization would better improve the translational potential of any molecular, biological, or drug studies.

## Conclusions

Our data provide strong evidence that the kinome of GBM does change when physiologically relevant glucose levels are utilized in comparison to the high glucose levels present in standard medias. As a proof of concept, we determined that PLCG1 peptide phosphorylation and efficacy of a PLC inhibitor were significantly altered in low glucose conditions. When considered in the broader context of drug screening, these data demonstrate the utility of high-throughput platforms that incorporate physiologically relevant microenvironments.

## Abbreviations

ACM5: Muscarinic acetylcholine receptor M5
GBM: Glioblastoma
NHA: Normal human astrocytes
PDX: patient derived xenografts
PLCG1: Phospholipase C, gamma 1
